# Platelets promote human macrophages-mediated macropinocytosis of *Clostridioides difficile*

**DOI:** 10.3389/fcimb.2023.1252509

**Published:** 2024-01-05

**Authors:** Angela María Barbero, Rodrigo Emanuel Hernández Del Pino, Federico Fuentes, Paula Barrionuevo, Virginia Pasquinelli

**Affiliations:** ^1^ Centro de Investigaciones Básicas y Aplicadas (CIBA), Universidad Nacional del Noroeste de la Provincia de Buenos Aires (UNNOBA), Buenos Aires, Argentina; ^2^ Centro de Investigaciones y Transferencias del Noroeste de la Provincia de Buenos Aires (CIT NOBA), UNNOBA-Universidad Nacional de San Antonio de Areco (UNSAdA)- Consejo Nacional de Investigaciones Científicas y Técnicas (CONICET), Buenos Aires, Argentina; ^3^ Instituto de Medicina Experimental (CONICET-Academia Nacional de Medicina), Buenos Aires, Argentina

**Keywords:** *C. difficile*, macrophages, platelets, macropinocytosis, uptake

## Abstract

*Clostridioides difficile* is the main causative agent of hospital-acquired diarrhea and the potentially lethal disease, *C. difficile* infection. The cornerstone of the current therapy is the use of antibiotics, which is not fully effective. The molecular mechanisms, inflammatory conditions and host-immune responses that could benefit the persistence or elimination of *C. difficile* remain unclear. Macrophages perform different ways of endocytosis as part of their immune surveillance functions and platelets, classically known for their coagulatory role, are also important modulators of the immune system. The aim of this study was to evaluate the endocytosis of vegetative *C. difficile* by human macrophages and the involvement of platelets in this process. Our results showed that both macrophages and platelets interact with live and heat-killed *C. difficile*. Furthermore, platelets form complexes with human monocytes in healthy donor’s fresh blood and the presence of *C. difficile* increased these cell-cell interactions. Using flow cytometry and confocal microscopy, we show that macrophages can internalize *C. difficile* and that platelets improve this uptake. By using inhibitors of different endocytic pathways, we demonstrate that macropinocytosis is the route of entry of *C. difficile* into the cell. Taken together, our findings are the first evidence for the internalization of vegetative non-toxigenic and hypervirulent *C. difficile* by human macrophages and highlight the role of platelets in innate immunity during *C. difficile* infection. Deciphering the crosstalk of *C. difficile* with immune cells could provide new tools for understanding the pathogenesis of *C. difficile* infection and for the development of host-directed therapies.

## 1 Introduction

*Clostridioides difficile* infection (CDI) is currently the leading cause of nosocomial diarrhea associated with the use of antibiotics (Brazier, 1998; Zhang et al., 2016). *C. difficile* is a Gram positive opportunistic pathogen that takes advantage of gut dysbiosis to establish colonization of the intestine, inducing tissue damage by toxin production. *C. difficile* is responsible for about 250,000 CDI cases and 13,000 deaths in the United States each year (Center for Disease Control and Prevention, 2019). Given the increase in the incidence and severity of CDI, the high rate of recurrence (>35%) and the appearance of hypervirulent strains, the Center for Control and Prevention in the United States has pointed this infection as an emergency (McDonald et al., 2007; CDC, 2012; Burke and Lamont, 2014; Balassiano et al., 2016; Elliott et al., 2017). Furthermore, there has been a large increase in community-acquired cases in the last decades, affecting individuals without risk factors typically associated with CDI (Legaria et al., 2003; Chitnis et al., 2013; Gupta and Khanna, 2014). Of importance, since the onset of the pandemic, approximately 72% of COVID-19 patients have been treated with broad-spectrum antibiotics. Therefore, the number of *C. difficile* infections, particularly recurrences, is expected to increase over time (EBR, 2020). All this together exhibit the urgent nature of this pathogen.

The immune response against *C. difficile* has been assessed before, however many aspects require a deeper understanding (Hernández Del Pino et al., 2020). Particularly, intestinal macrophages demand constant renewal from circulating monocytes, shaping the composition of the macrophage compartment in the intestinal mucosa when infection, inflammation or trauma alter tissue homeostasis (Bain and Mowat, 2014b; Na et al., 2019). Intestinal macrophage populations are heterogeneous and display different functions. Both anti-inflammatory and pro-inflammatory macrophages of the gut wall derive from blood monocytes that enter the mucosa and mature locally (Bain and Mowat, 2014a; Bain and Mowat, 2014b). In experimental CDI models, macrophages produce pro-inflammatory cytokines and chemokines, as well as show expression of certain receptors and molecules that indicate macrophages activation (Vohra and Poxton, 2012; Collins et al., 2014). Besides, the interaction and internalization of *C. difficile* spores and toxins has been observed in monocytes, macrophages or epithelial cells (Solomon et al., 2005; Modi et al., 2011; Paredes-Sabja et al., 2012; Chen et al., 2020). To our knowledge, the internalization of vegetative bacteria has not been demonstrated yet.

Platelets have aroused great interest in recent years, proving to be key participants in numerous immunological processes. Beyond their central roles in thrombosis and hemostasis, platelets play multiple roles in host defense against infection, interacting with bacteria, protozoa, fungi and viruses and showing anti-microbial properties (Klinger, 1997; Yeaman, 1997; Lam et al., 2015). Platelets can also interact with innate and adaptive immune cells promoting neutrophils activation, cytokines secretion by monocytes, leukocytes migration to the site of inflammation, B cells differentiation and immunoglobulin classes switching (Klinger, 1997; Yeaman, 1997; Trzeciak-Ryczek et al., 2013; Maouia et al., 2020; Scherlinger et al., 2023). In the context of CDI, severe infection could lead to sepsis and extra-intestinal infection (Lowenkron et al., 1996; Libby and Bearman, 2009; Steele et al., 2012; Abid and Bischof, 2019; Olsen et al., 2023). Moreover, inflammation in the gut can induce platelet activation and migration (Gaertner et al., 2017; Shannon, 2021; Trivigno et al., 2023), then platelets attached to the activated endothelium promote monocyte migration (Huilcaman et al., 2022; Trivigno et al., 2023). Beneficial or detrimental roles related to clinical symptoms have been reported for platelets counts during CDI (Pant et al., 2009; Byrn et al., 2012; Lee et al., 2014; Yan et al., 2017; Mihăilă et al., 2019; Nseir et al., 2019; Allegretti et al., 2020; Phanchana et al., 2020; Zhao et al., 2020; Buchrits et al., 2021). Nevertheless, their modulatory functions on immune responses against *C. difficile* have not been addressed.

Our findings provide new insights into the role of macrophages and platelets during CDI, which could be useful for the development of new therapeutic approaches in search of replacing antibiotics or as treatment adjuvants.

## 2 Materials and methods

### 2.1 Ethics statement and human samples

This research was carried out in accordance with the Helsinki declaration (2013) in agreement with the Ethics Committee of UNNOBA (COENOBA). Human blood from adult healthy donors was collected after obtaining written informed consents. The male/female group distribution was 1/2 and the median age was 36 yr.

### 2.2 Bacteria

*In vitro* infection of cells was performed with vegetative live non-toxigenic CD160 (NR-43516, BEI Resources) or hypervirulent NAP1/BI/027 (Sanitary Bacteriology Service INEI-ANLIS, Dr. Carlos G. Malbrán, Argentina) *C. difficile* strains. *C. difficile* was cultured at 37°C under anaerobic and dark conditions (anaerobic jars and envelopes from Mitsubishi Gas Chemical Company, Inc, Japan) in CHROMagar™ *C. difficile* and then in BHI Broth for 48h and 72h, respectively. NAP1/BI/027 strain was additionally inactivated by heat treatment (*CD*H). *C. difficile* culture broth was centrifuged at 25000 g for 10 minutes at 4°C. The bacteria pellet was washed with 1X PBS and inactivated at 95°C for 30 minutes. The antigenic content was quantified at 600 nm and adjusted to an OD of 1 for parity between experiments. NAP1/BI/027 secretome was also employed in some experiments. *C. difficile* culture supernatant was centrifuged, filtered with 0.22 μm membrane pore and protein content was determined by Micro BCA Protein Assay (Thermo Fisher Scientific Inc.).

For uptake assays, CD160, NAP1/BI/027 and *CD*H were coupled to FITC (Fluorescein isothiocyanate isomer I, Sigma Aldrich, St. Louis, MO, EE. UU) in carbonate buffer (NaHCO_3_ 0,1M pH=9) for 2h at 37°C. Excess of FITC was removed by washing with 1X PBS and bacterial viability was evaluated (**Supplementary Figure 1D**).

### 2.3 Whole blood

Blood samples from healthy donors were collected into plastic tubes containing 3.8% sodium citrate in a blood:sodium citrate proportion of 10:1. Whole blood (50 μl) was cultured for 2h in the presence or absence of 0.5 × 10^6^ *CD*H bacteria to evaluate the formation of monocytes-platelets complexes.

### 2.4 Macrophages

Peripheral blood mononuclear cells (PBMCs) from the whole blood samples of healthy donors were isolated by centrifugation over Ficoll-Hypaque (GE Healthcare, Chicago, IL, USA). Monocytes were purified from PBMCs by CD14 positive magnetic selection (Miltenyi Biotec) according to the manufacturer’s instructions. In all cases, the purity of the isolated cells was over 95%.

0.5 x 10^6^/ml CD14 positive-selected monocytes were cultured at 37°C and 5% CO2 for 2h in absence of FBS to promote adherence. Non-adherent cells were removed and adherent cells were cultured for additional 16-18h in complete media as described before (Barbero et al., 2021) (**Supplementary Figure 1A**).

Monocyte-derived macrophages were stimulated or infected with 1 × 10^6^ or 1 × 10^7^ bacteria of *CD*H, *CD*H FITC, live CD160 FITC, or live NAP1/BI/027 FITC for 1h or 24h. For some experiments, cells were incubated in the presence or absence of washed platelets. To evaluate endocytic pathways, different inhibitors were added to the cell cultures 30 minutes previous to *CD*H stimulation: cytochalasin B (5 µg/ml), cytochalasin D (5 µg/ml), colchicine (1 µg/ml), vincristine (10 µg/ml), nystatin (100 U/ml), amiloride (100 µg/ml) or bafilomycin A (0,1 µM).

### 2.5 Platelets

Blood samples were collected into plastic tubes containing 3.8% sodium citrate in a blood:sodium citrate proportion of 10:1. Platelet-rich plasma (PRP) was obtained by blood sample centrifugation. To avoid leukocyte contamination, only the top 75% of the PRP was collected and centrifuged. Platelets were then washed with a buffer containing sodium citrate, citric acid, glucose and sodium chloride as reported before (Ali et al., 2017) and were resuspended in pre-warmed RPMI.

Washed platelets (WP) were then cultured for 2h in the presence or absence of *CD*H, *CD*H FITC, live CD160 FITC or live NAP1/BI/027 FITC at a platelet:bacteria ratio of 1:1 or 1:10 or used in co-culture experiments at macrophage:bacteria:platelet ratios of 1:1:10, 1:1:100 or 1:10:100 for 1h or 24h.

### 2.6 Flow cytometry

For whole blood experiments, the samples were fixed and erythrocytes were lysed with BD FACS^®^ Lysing Solution (BD Biosciences) for 15 minutes at room temperature after staining.

In the case of WP cultures, the cells were centrifuged at 1500g for 10 minutes with no break. For monocyte-derived macrophages or cells from macrophages-WP co-cultures, cell collection was done by extensive washing with FACS buffer (1X PBS – 2% FBS) and 5 minutes’ centrifugation at 800 g. Samples were stained for 30 minutes at 4°C, washed and fixated with 1% PFA.

Fluorochrome-conjugated antibodies against CD14 (BioLegend^®^) or CD61 (BD Pharmigen™) were used for immune staining. A viability dye (Fixable Viability Dye eFluor™ eBioscience™ 780) was employed to exclude dead cells from analysis.

All samples were acquired on a FACSCanto II flow cytometer (BD Biosciences) and data analysis was performed using FlowJo 7.6.2 (Tree Star Inc., OR, USA).

### 2.7 Fluorescence and confocal microscopy

2 × 10^5^ macrophages/well were cultured in chamber-slides (Nunc) as described before (Barbero et al., 2021). Cells were stimulated with *CD*H FITC and/or WP at the ratios indicated above in the presence or absence of endocytosis inhibitors.

Cells were fixed with 2% PFA for 20 minutes, washed with saline solution and permeabilized (1X PBS, 10% FBS and 0.5% saponin). Afterwards, cells were incubated with specific PE mouse anti-human CD61 (BD Pharmigen™) and Alexa Fluor 647 mouse anti-human CD14 (BioLegend^®^) antibodies. After washing, Flash Phalloidin Red 594 (BioLegend^®^) was added to stain F-actin and DAPI (Life Technologies) was employed as nuclear counterstaining.

Slides were mounted with PolyMount (Polysciences) and analyzed using trinocular fluorescence microscope Axio Imager.A2 (Carl Zeiss, Germany) or FV-1000 confocal microscope with an oil-immersion Plan Apochromatic 60X NA1.42 objective (Olympus). For quantification, at least 100 cells per field and 5 fields per treatment were analyzed in three independent experiments. Micrographs were taken in a blind manner. The obtained images were processed with FIJI software (open source, version ImageJ 2.0.0-rc-69/1.52p/java 1.8.0_172).

### 2.8 Statistical analysis

All assays were carried out at least three times in independent experiments. Representative examples are shown for flow cytometry histograms and confocal/fluorescence micrographs. Friedman test or Kruskal-Wallis test with corrected Dunn’s *post hoc* test were used for comparison between experimental groups. Mann-Whitney test was used to analyze differences between two groups of unpaired samples and Wilcoxon Rank Sum test or paired t test for paired samples.

Data was analyzed using GraphPad Prism 8.0.1 software (San Diego, CA, USA). p values < 0.05 were considered significant.

## 3 Results

### 3.1 *C. difficile* is internalized by human macrophages

We addressed the uptake of vegetative *C. difficile* form, which is necessary to produce toxins and spores in the gut. To identify interactions between macrophages and heat inactivated *C. difficile* (*CD*H) by flow cytometry and by confocal microscopy, *CD*H was coupled to FITC. *CD*H positive staining (**Supplementary Figures 1B, C**) and macrophages viability for all the bacteria amounts employed (**Supplementary Figure 2A**) were corroborated.

**Figure 1:**
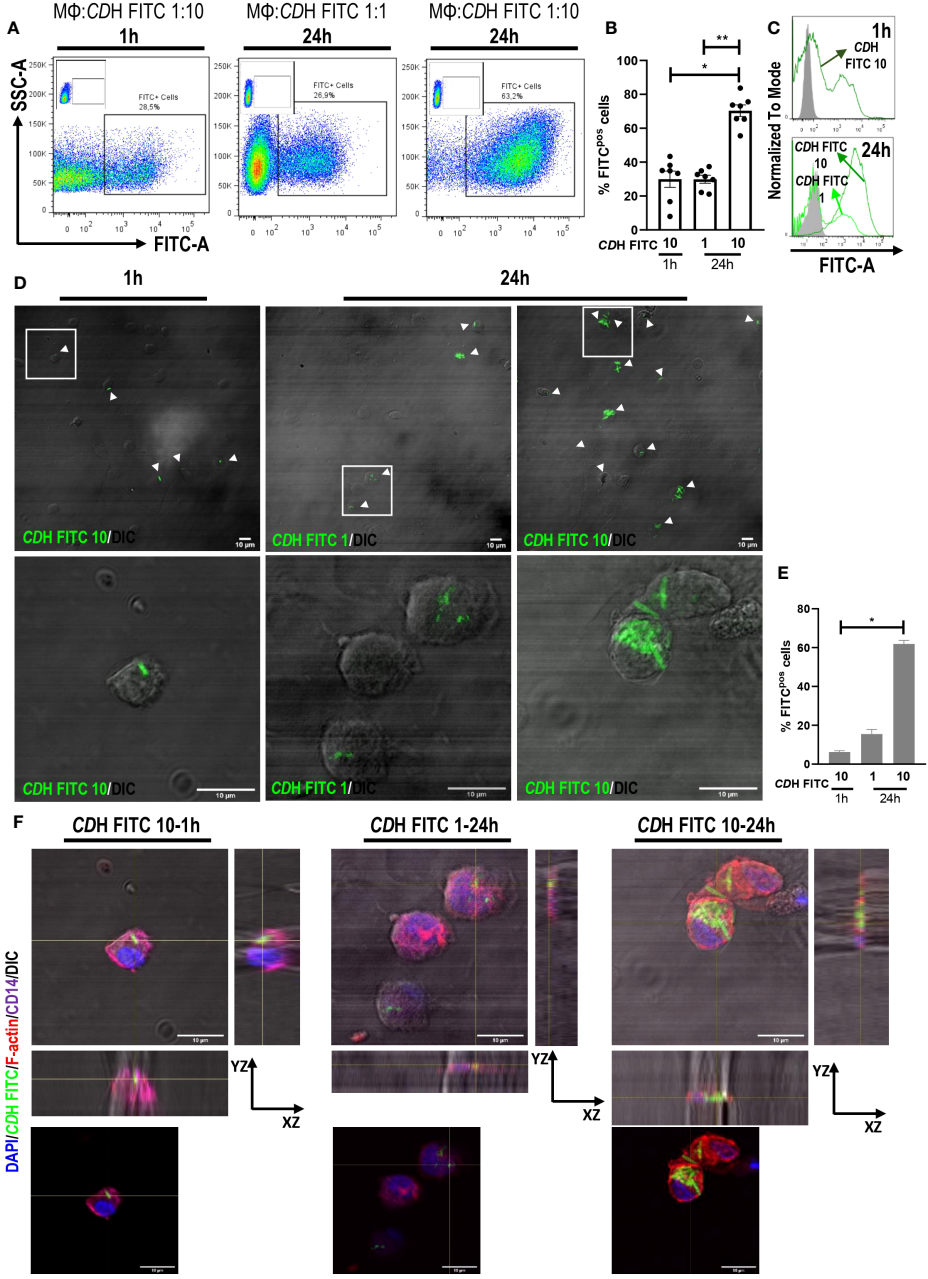
Internalization of *C. difficile* by human macrophages. Monocyte-derived macrophages (MΦs) from Healthy Donors (HD) were stimulated with heat-inactivated *C. difficile* coupled to FITC (*CD*H FITC). The ratios of MΦs:*CD*H FITC tested were 1:10 for 1h and 1:1 and 1:10 for 24h assays. **(A)** Representative dot plots, **(B)** percentage of FITC positive cells (macrophages internalizing *CD*H) and **(C)** representative histograms of flow cytometry experiments are shown. MΦs stimulated with unstained *CD*H were used as controls (gray histograms). **(D)** Representative micrographs (white boxes correspond to the magnified images shown in the bottom panels), **(E)** percentage of FITC positive cells and **(F)** orthogonal views (same magnification as in **(D)** showing the internalization of *CD*H FITC evaluated by confocal microscopy after cell fixation. White arrowheads point macrophages internalizing *CD*H. DAPI (nuclei) is shown in blue, Phalloidin (F-actin) in red and CD14 (macrophages) in purple. DIC: differential interference contrast. Scale bar: 10 μm **(A–C)** correspond to seven individual donors in seven independent experiments, **(D–F)** correspond to three individual donors in three independent experiments. **(B, E)** Bars represent the mean ± SEM. **(B)** Each dot corresponds to an individual healthy donor. **(B)** Friedman test, **(E)** Kruskal-Wallis test. *p<0.05, **p<0.01.

We found that about 29% of human macrophages interacted with bacteria both at 1h (macrophages:*CD*H 1:10) or 24h (macrophages:*CD*H 1:1) of culture (**Figures 1A–C**). Likewise, 70% of macrophages interacted with *CD*H FITC when cultured for 24h at a macrophages to *CD*H ratio of 1:10 (**Figures 1A–C**), suggesting a time- and load-dependent uptake of *CD*H by macrophages. When analyzing confocal micrographs, *CD*H FITC was observed within macrophages in all experimental conditions (**Figures 1D–F**). The intracellular localization was confirmed by orthogonal views sectioning (**Figure 1F**) and Z-stacks reconstructions (**Supplementary Videos SV1–3**). In addition, the amount of interaction between macrophages and *CD*H FITC resembled flow cytometry results (**Figure 1E**). Interestingly, infection of macrophages with live CD160 and NAP1/BI/027 strains or incubation with CDH plus NAP1/BI/027 secretome induced similar endocytic levels compared to CDH (**Supplementary Figure 4B**).

Altogether, our results demonstrate for the first time the internalization of vegetative *C. difficile* by human macrophages.

### 3.2 Platelets interact with *C. difficile*, monocytes and macrophages

**Figure 2:**
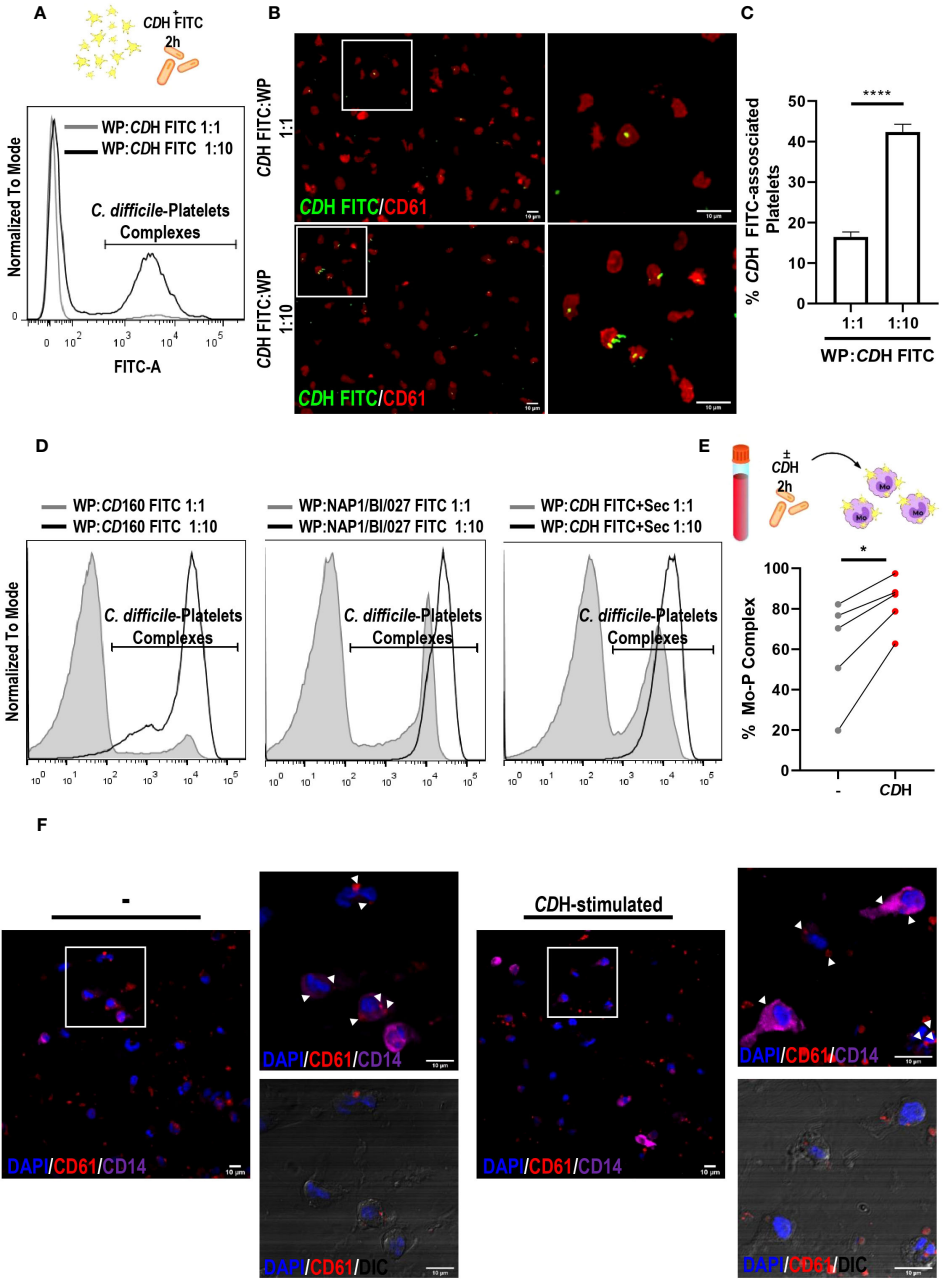
Interaction of platelets with *C. difficile*, monocytes and macrophages. **(A–C)** Washed Platelets (WP) from Healthy Donors (HD) were incubated with heat-inactivated *C. difficile* coupled to FITC (*CD*H FITC) at ratios 1:1 and 1:10 for 2h. *CD*H FITC-WP complexes were detected by flow cytometry **(A)** and fluorescence microscopy **(B, C)** using anti-CD61 PE antibody. **(A, B)** are representative experiments. **(B)** Images on the right are magnifications corresponding to white boxes on the left. **(C)** Quantification of *CD*H FITC-associated platelets from fluorescent micrographs is shown. **(D)** WP from HD were incubated with live CD160 and live NAP1/BI/027 *C. difficile* strains coupled to FITC and with *CD*H FITC plus NAP1/BI/027 secretome (*CD*H FITC+Sec) for 2h. In all cases, WP:bacteria ratios of 1:1 and 1:10 were tested and *C. difficile*-WP complexes were detected by flow cytometry. Representative histograms are shown. **(E)** Whole peripheral blood from HD was cultured in the presence or absence of *CD*H for 2h. Monocytes (Mo)-WP complexes were measured by flow cytometry using anti-CD14 Alexa Fluor 647 and anti-CD61 PE antibodies. **(F)** Macrophages (MΦs) and WP from HD were co-cultured in the presence or absence of *CD*H for 1h. MΦs-WP complexes were assessed by confocal microscopy after fixation, permeabilization and staining. The white boxes correspond to the magnified images on the right (representative experiment). White arrowheads point WP inside MΦs. DAPI (nuclei) is shown in blue, CD61 (platelets) in red and CD14 (macrophages) in purple. DIC: differential interference contrast. Scale bar: 10 μm **(A, C, F)** correspond to three individual donors in three independent experiments, **(D)** corresponds to four independent experiments, **(E)** corresponds to five individual donors in five independent experiments. **(C)** Bars represent the mean ± SEM. **(E)** Each dot corresponds to an individual healthy donor. **(C)** Mann-Whitney test, **(E)** Wilcoxon test. *p<0.05, ****p<0.0001.

In the context of *C. difficile* infection (CDI), the role of platelets in the outcome of the disease is still controversial. Here, we first evaluated platelets-*C*. *difficile* FITC interaction. We observed that both live NAP1/BI/027 and CD160 strains and *CD*H FITC were associated to platelets. Moreover, these associations were increased in the presence of a greater amount of bacteria as shown by flow cytometry and fluorescence microscopy (**Figures 2A–D**). We then tested the formation of platelets–monocytes complexes in whole blood from healthy donors. Whole blood was incubated for 2h with or without *CD*H and then stained with anti-CD14 and anti-CD61 antibodies (**Supplementary Figure 2B**). Flow cytometric analysis showed that *CD*H induced the establishment of complexes, as the percentage of monocytes bound to platelets was increased when compared to controls (**Figure 2E**). In addition, we co-cultured different proportions of platelets with macrophages that were stimulated or not with *CD*H. No differences in platelets-macrophages complexes formation were found in the presence of *CD*H after 24h, even when the macrophage:platelet ratio was increased 10-fold (macrophages:platelets, 1:10 vs 1:100) (**Supplementary Figure 4A**). We neither observed changes in platelets bounding to macrophages when the co-cultures were done for 1h using confocal microscopy (**Figure 2F**). However, we did detected platelets inside the macrophages (**Figure 2F**). We also noted that platelets showed a great ability to interact with macrophages both at 1h and 24h of co-culture since the majority of the phagocytes had associated platelets (**Figure 2F** and **Supplementary Figure 4A**).

In brief, these results show that platelets can interact with both immune cells and *C. difficile*.

### 3.3 *C. difficile* uptake is promoted by platelets

We designed two different approaches to evaluate platelets role in the uptake of *C. difficile*: 1- Platelets, *CD*H and macrophages were cultured all three together for 24h or 2- Platelets were pre-incubated with *CD*H for 2h and added to macrophages culture for additional 22h. Likewise, different macrophages:*C. difficile*:platelets ratios were tested.

**Figure 3:**
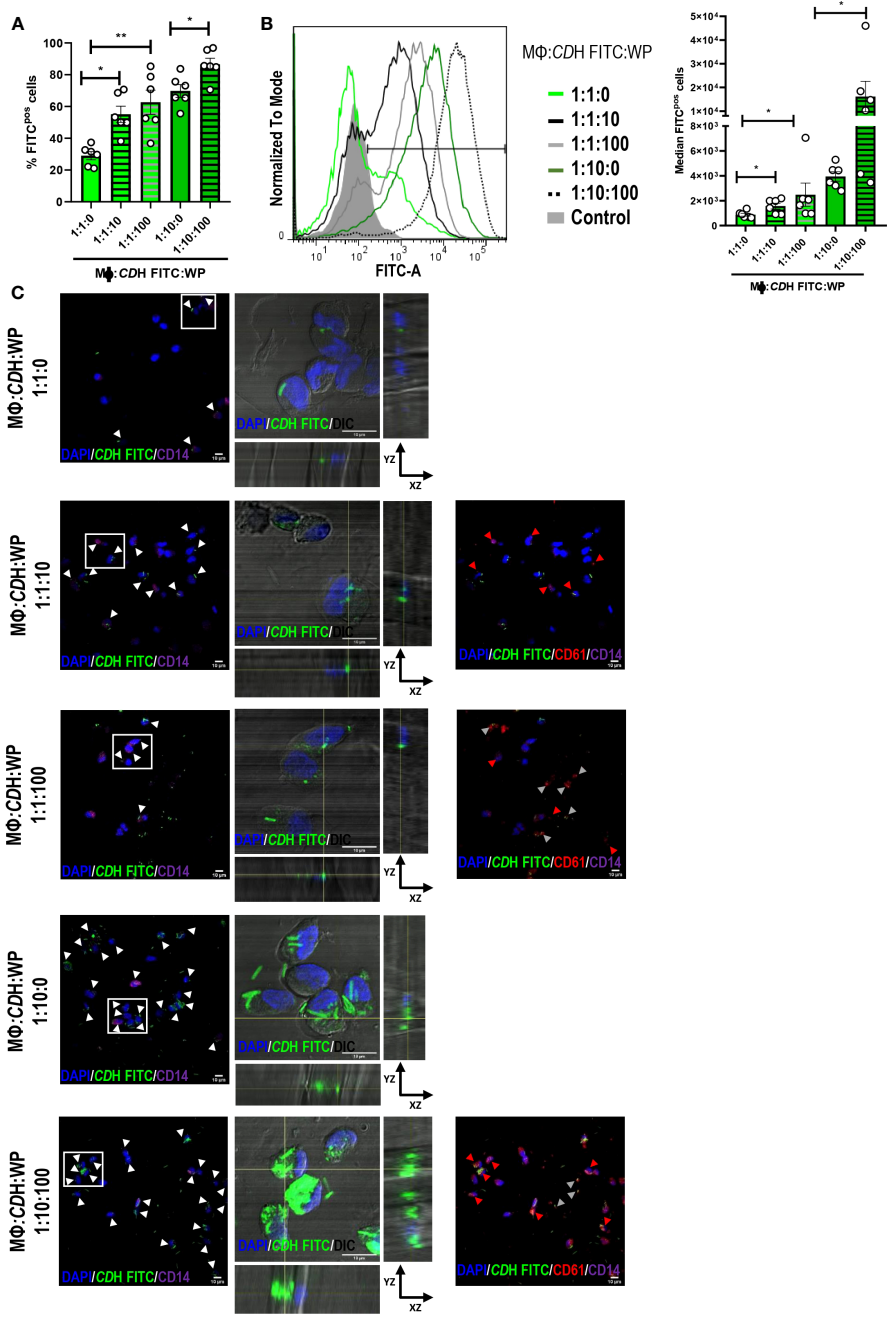
Role of platelets in *C. difficile* uptake. **(A, B)** Macrophages (MΦs) from Healthy Donors (HD) were stimulated with heat-inactivated *C. difficile* coupled to FITC (*CD*H FITC) in the presence or absence of Washed Platelets (WP) in ratios of 1:1:10, 1:1:100 and 1:10:100 (MΦs:*C. difficile*:WP) for 24h. The percentage of FITC positive cells **(A)** and the median intensity of fluorescence **(B)** were evaluated by flow cytometry. **(B)** Representative histograms (left) and the intensity quantification (right) are shown. In all cases comparisons were done against the experimental conditions without platelets (1:1:0 or 1:10:0). **(C)** MΦs: *CD*H FITC: WP cultures were performed as in A-B and the internalization capacity was evaluated by confocal microscopy. MΦs and WP were detected by direct staining with anti-CD14 Alexa Fluor 647 and anti-CD61 PE antibodies after fixation and permeabilization of the cells. DAPI was used as nuclear counterstaining. The white boxes correspond to the magnified images on the right (orthogonal views showing internalized *CD*H FITC in macrophages (DIC/DAPI)). In the panoramic micrographs, white arrowheads point macrophages (CD14/DAPI) internalizing *CD*H, red arrowheads point WP (CD61) and gray arrowheads indicate not internalized *CD*H bounded to platelets (CD61, red). DAPI (nuclei) is shown in blue, CD61 (platelets) in red and CD14 (macrophages) in purple. DIC: differential interference contrast. Scale bar: 10 μm. **(A, B)** correspond to six individual donors in six independent experiments, **(C)** correspond to three individual donors in three independent experiments. **(A, B)** Bars represent the mean ± SEM. Each dot corresponds to an individual healthy donor. **(A, B)** Friedman (1:1:10, 1:1:100 vs 1:1:0) and Wilcoxon (1:10:100 vs1:10:0) tests. *p<0.05, **p<0.01. Control= MΦs stimulated with unstained *CD*H.

*CD*H FITC-stimulated macrophages showed greater endocytic capacity when platelets were present in the cell culture (**Figures 3A, B**) as measured by flow cytometry. The highest percentage of FITC positive macrophages (macrophages internalizing *CD*H) was observed after *CD*H FITC stimulation in combination with platelets at the 1:10:100 proportion (**Figure 3A**). Moreover, the median intensity of fluorescence was also significantly increased in the presence of platelets in all the assessed ratios (**Figure 3B**), indicating that not only the number of *C. difficile-*internalizing macrophages increases, but also the amount of bacteria per phagocyte. Similar results were observed when platelets and *CD*H were pre-incubated for 2h (**Supplementary Figures 3A, B**). When evaluating confocal micrographs (**Figure 3C**), the majority of those macrophages that had internalized the bacteria presented associated platelets, confirming that platelets induce *C. difficile* uptake (**Figure 3C**, red arrowheads). We also noted that not internalized *CD*H was bound to platelets (**Figure 3C**, grey arrowheads). Anew, similar results were found in the pre-incubation system (**Supplementary Figure 3C**).

Taken together, these results show that human macrophages enhance their internalizing potential against *C. difficile* in the presence of platelets.

### 3.4 Macrophages explore macropinocytosis for *C. difficile* uptake

**Figure 4:**
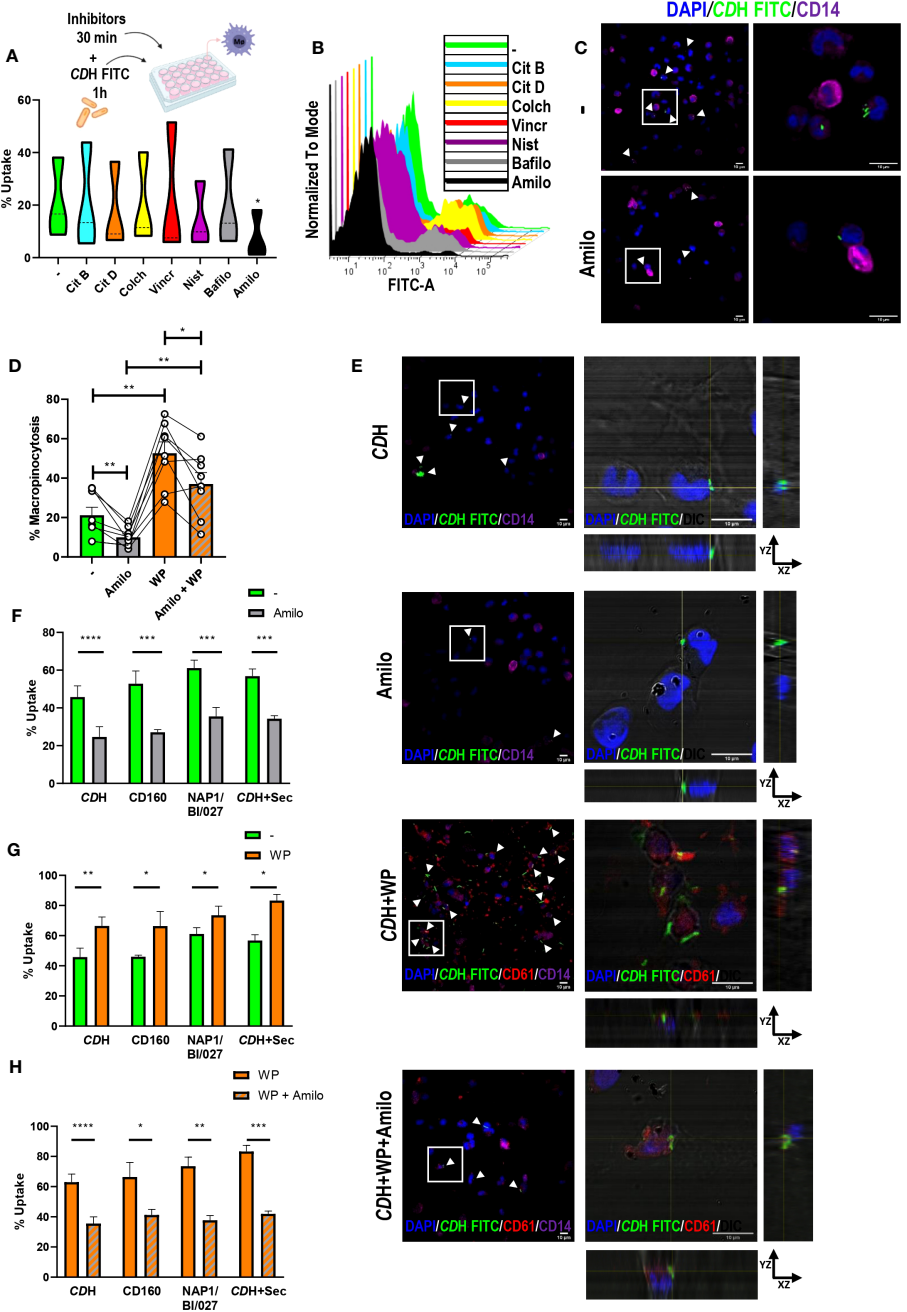
Evaluation of endocytic pathways against *C. difficile*. A) Macrophages (MΦs) from Healthy Donors (HD) were cultured with endocytosis inhibitors (Citochalasin B (Cit B, actin polymerization inhibitor), Citochalasin D (Cit D, inhibitor of actin polymerization by ATP hydrolysis), Colchicine (Colch, microtubules polymerization inhibitor), Vincristine (Vincr, microtubules polymerization inhibitor), Nystatin (Nist, inhibitor of caveolae/lipid mediated endocytosis), Bafilomycin (Bafilo, autophagy inhibitor) or Amiloride (Amilo, macropinocytosis inhibitor)). After 30min, heat-inactivated *C. difficile* coupled to FITC (*CD*H FITC) was added for 1h. *CD*H FITC internalization was assessed by flow cytometry **(A, B)** and confocal microscopy **(C)**. **(A)** Percentage of FITC positive cells (uptake). **(B)** Representative histograms. **(C)** Confocal micrographs of untreated (-) or Amiloride treated (Amilo) MΦs obtained after cell fixation, staining with anti-CD14 Alexa Fluor 647 antibody (macrophages) and DAPI (nuclei) counterstaining. The white boxes correspond to the magnified images on the right. White arrowheads point internalized *CD*H FITC. **(D)** MΦs were stimulated with *CD*H FITC in the presence or absence of Washed Platelets (WP) (ratio 1:10:100) for 1h. Amiloride was added 30 minutes before to block macropinocytosis. Percentage of macropinocytic cells was measured by flow cytometry. **(E)** Cells were fixated, permeabilized and stained with anti-CD14 Alexa Fluor 647 (macrophages) and anti-CD61 PE (platelets) antibodies. DAPI (nuclei) was employed as counterstain and confocal images were obtained. The white boxes correspond to the magnified images on the right (orthogonal views showing platelets (CD61) and internalized *CD*H FITC in macrophages (DIC/DAPI)). In the panoramic micrographs, white arrowheads point macrophages internalizing *CD*H. DAPI (nuclei) is shown in blue, CD61 (platelets) in red and CD14 (macrophages) in purple. DIC: differential interference contrast. Scale bar: 10 μm. **(F–H)** MΦs were stimulated with *CD*H FITC or *CD*H FITC plus NAP1/BI/027 secretome (*CD*H FITC+Sec) or infected with live CD160 FITC (non-toxigenic) or NAP1/BI/027 FITC (hypervirulent) *C. difficile* strains as stated in **(D)**. **(A–C, E)** correspond to three individual donors in three independent experiments, **(D)** correspond to nine individual donors in 9 independent experiments. **(F–H)** correspond to four donors in independent experiments. **(A)** Violin plots, **(D, F–H)** Bars represent the mean ± SEM. **(D)** Each dot corresponds to an individual healthy donor. **(A, D)** Friedman test. **(F–H)** 2-way ANNOVA *p<0.05, **p<0.01, *** p<0.001, **** p<0.0001.

To deduce the route of entry involved in *C. difficile* uptake we used selected inhibitors for different endocytic pathways (**Table 1**). Since *C. difficile* presents length variations that can reach up to 12 µm (Lynch et al., 2013; Ransom et al., 2014; Metzendorf et al., 2022), no inhibitors of clathrin-mediated uptake were used [this mechanism shows an upper size limit for internalization of approximately 200 nm approximately 200 nm (Rejman et al., 2004; Rennick et al., 2021; Sousa De Almeida et al., 2021)]. Macrophages were pre-incubated for 30 minutes with cytochalasin B, cytochalasin D, colchicine, vincristine, nystatin, bafilomycin A or amiloride before *CD*H FITC was added to the cell culture for 1h. We observed that only amiloride was able to reduce the percentage of FITC positive macrophages compared to the control (**Figures 4A, B**), suggesting that macropinocytosis helps macrophages to engulf *C. difficile*. On the other hand, neither autophagy, caveolae/lipid-mediated endocytosis, or microtubule polymerization appear to be involved in *C. difficile* internalization in our culture model (**Figures 4A, B**). We confirmed that amiloride reduced the number of cells that internalized *C. difficile* (**Figure 4C**, white arrowheads) by confocal microscopy. Moreover, for those FITC-positive cells, a decrease in the number of bacilli inside the cells was observed (**Figure 4C**, magnifications). Therefore, in agreement with our previous findings, when macropinocytosis was inhibited, macrophages reduced their ability to internalize *C. difficile* (**Figure 4C**).

**Table 1:** Endocytosis inhibitors information.

Inhibitor	Mechanism involved
**Cytochalasin B**	Fungal toxin. Disorganization of the extracellular fibronectin filaments following the alteration of the microfilament network. Inhibition of actin filament polymerization through binding to the fast-growing (barbed) end of F-actin filaments.
**Cytochalasin D**	Fungal toxin. Disruption of network organization, increasing the number of actin filament ends and leading to the formation of filamentous aggregates or foci. Increment in the initial rate of polymerization and drop in the final extent of the reaction.
**Colchicine**	Alkaloid of *Colchicum autumnale* plant. Disappearance of cytoplasmic microtubules and disorganization of the Golgi complex.
**Vincristine**	Alkaloid of the *Madagascar periwinkle*. Destabilization of microtubules affecting intracellular trafficking of organelles (e.g., endosomes, lysosomes, and autophagosomes).
**Nystatin**	Polyene from *Streptomyces noursei*. Inhibition of caveolae/lipid-mediated endocytosis as it binds sterols and disrupts the formation of caveolae.
**Bafilomycin**	Macrolide from *Streptomyces griseus.* Inhibition of autophagosome-lysosome fusion and the activity of autophagic flux.
**Amiloride**	Pyrazine. Inhibitor of Na+/H+ exchangers, lowering submembranous pH and preventing macropinocytosis.

Additionally, we evaluated the effect of amiloride treatment in the induction of *C. difficile* uptake mediated by platelets. Macrophages were cultured with live NAP1/BI/027 FITC, live CD160 FITC, *CD*H FITC or *CD*H FITC plus NAP1/BI/027 secretome for 1h after being pre-exposed to amiloride, platelets or the combination of platelets plus amiloride for 30 minutes. As detected by flow cytometry and confocal microscopy, platelets were capable of improving *CD*H FITC internalization also at short times of co-culture (**Figures 4D, E** and **Supplementary Figure 4C**). On the other hand, amiloride significantly reduced these levels both when added in combination with platelets or alone (**Figures 4D, E** (white arrowheads) and **Supplementary Figure 4C**). Similar results were observed for live non-toxigenic and hypervirulent *C. difficile* as well as for *CD*H FITC plus NAP1/BI/027 secretome (**Figures 4F-H**). Overall, these findings show that macropinocytosis allows *C. difficile* internalization by human macrophages and that platelets participate in this process (**Supplementary Figure 4D**).

Our results show that *CD*H is a valuable tool for *in vitro* studies assessing innate responses against *C. difficile*. Beyond understanding the immune responses against *C. difficile*, working with killed or attenuated pathogens may lead to the development of prevention strategies, vaccines, or even new host-directed therapies. Moreover, inactivated bacteria provide a constant and standardized source of antigens which is essential to perform reproducible experiments and compare results between different assays or laboratories.

Altogether, our findings suggest that the mechanism described in this work could be widely used by different strains of the *C. difficile* family regardless of the presence of toxins and that endocytosis is probably induced by antigens that are expressed in both live and dead bacteria. Moreover, this is the first report describing platelets as active participants in innate immunity against *C. difficile* boosting macrophages-mediated uptake.

## 4 Discussion

*Clostridioides difficile* infection (CDI) is prevalent in hospitalized patients, and despite prevention activities, CDI remains a life-threatening disease, highlighting the need for new treatment approaches and a better understanding of host-pathogen crosstalk.

It is now well-known that innate immunity is a double-edged sword during CDI, since it is crucial for inducing intestinal inflammation but also for resolving infection (Hernández Del Pino et al., 2020; Nibbering et al., 2021). Regarding macrophages, most of the reports are based on cell lines or murine models and evidence the activation of macrophages in response to *C. difficile*. Macrophages produce proinflammatory MIP-1α, MIP-2, MCP, IL-1β, IL-6, TNF-α, IL-12p40, and IL-12p70 when stimulated with *C. difficile* antigens (e.g. surface layer proteins, flagella, HSP42, and HSP60) or live bacteria (Vohra and Poxton, 2012; Collins et al., 2014; Saad et al., 2023). Likewise, these phagocytes up-regulate TLR2, TLR4, CD40, CD80, and MHCII expression (Collins et al., 2014). Moreover, inflammasome activation via TLR2-ATP-P2X7-Caspase 1 has been proposed as a beneficial mechanism to control bacterial load (Liu et al., 2018).

In our work we employed human monocyte-derived macrophages to address the endocytic capacity of these cells. Despite the fact that the intracellular localization of *C. difficile* has not been directly demonstrated yet, it has been proposed that phagocytosis of the bacteria or its toxins is essential to activate macrophages (Liu et al., 2018). *C. difficile* spores can be internalized by murine macrophages but they manage to survive and avoid their clearance (Paredes-Sabja et al., 2012). *C. difficile* toxin A can remodel cell membranes and gain access to interact with TLR9 in epithelial cells (Chen et al., 2020). Taking into account this information and the importance of the vegetative bacteria to induce pathogenesis at local level, we performed our experiments with the vegetative form of the *C. difficile* hypervirulent epidemic strain BI/NAPI/027 and the non-toxigenic CD160 strain. We found that human macrophages internalized *C. difficile* both at 1 or 24h of *in vitro* culture, showing greater uptake levels when incubated with higher amounts of bacteria. Our work is one of the first using different live *C. difficile* strains in normoxic *in vitro* models. We stained both live and inactivated *C. difficile* with FITC using a short time and few-steps protocol, guaranteeing bacterial viability. We believe that this could constitute a powerful tool for future research about the role of immune and non-immune cells in disease conditions.

The partnership between macrophages and platelets has been addressed in arteries pathologies (Mansilla et al., 2008; Mehrpouri et al., 2019; Huilcaman et al., 2022) and infections (Honda et al., 2009; Feng et al., 2014; Wong et al., 2016; Ali et al., 2017; Carestia et al., 2019; Rossaint et al., 2021). In the context of CDI, the studies are scarce and generally evaluate the importance of the platelet count in the severity of the disease. Some reports propose that an increased platelets number is associated with a better prognosis (Byrn et al., 2012; Lee et al., 2014; Yan et al., 2017; Allegretti et al., 2020; Zhao et al., 2020), while other studies associate this to worse outcomes (Albrich and Rimland, 2007; Pant et al., 2009; Mihăilă et al., 2019; Nseir et al., 2019; Phanchana et al., 2020; Buchrits et al., 2021). In severe cases of *C. difficile* infection, the intestine fails in its function as a barrier to both intestinal bacterial and toxins, allowing them to reach the portal circulation and developing extra-intestinal CDI (Lowenkron et al., 1996; Libby and Bearman, 2009; Steele et al., 2012; Abid and Bischof, 2019; Olsen et al., 2023). Moreover, vascular changes ligated to *C. difficile*-induced inflammation have been demonstrated as well as increased colonic vascular permeability and angiogenesis (Kurose et al., 1994; Huang et al., 2019). Platelets attached to the activated endothelium promote the migration of monocytes and can also modulate the composition of the intimal layer of blood vessels which favors monocytes migration (Huilcaman et al., 2022; Trivigno et al., 2023). With the end to get a better understanding of platelets role on human macrophages responses during CDI, we assessed the intercommunication of platelets with *C. difficile* and innate immune cells.

Platelets can directly sense microorganisms (Houlihan and Copley, 1946). Platelets can migrate to sites of infection and aggregate with the infiltrating bacteria, in addition to collaborating with the immune system (Gaertner et al., 2017; Shannon, 2021; Trivigno et al., 2023). The ability of platelets to recognize, collect and/or bound pathogens has been described for *E. coli* (Gaertner et al., 2017; Palankar et al., 2018)*, S. aureus* (Gaertner et al., 2017)*, L. monocytogenes* (Gaertner et al., 2017)*, B. abortus* (Trotta et al., 2018), and even retroviruses (Zucker-Franklin et al., 1990). Then, bacteria sensing by platelets is not restricted to a particular type of microorganism, since both live and inactivated, Gram positive and Gram negative, opsonized or not bacteria can be recognized. In line with these works, we evidenced a direct interaction of platelets with *C. difficile*. Platelets also act as covercytes, spreading on the foreign microorganism to cover and seal it (White, 2005). No specific molecule for the interaction between platelets and bacteria has been identified. Rather, it has been proposed that physical forces that help the platelets become “sticky” are responsible for platelet-bacteria interaction. Accordingly, we observed changes in platelets shape which could be related to actomyosin system that shapes the spreading and contraction dynamics of platelets. Besides, we described the establishment of monocytes-platelets complexes in fresh blood, which were induced in the presence of the bacteria. In accordance, monocytes-platelets crosstalk has also been observed for tetanus toxoid (Gudbrandsdottir et al., 2013) and *B. abortus* (Trotta et al., 2018), in sepsis (Gawaz et al., 1995), in Dengue patients (Hottz et al., 2014) and in non-infectious contexts (Weyrich et al., 1996; Weyrich et al., 2005). We also showed that platelets formed complexes with macrophages when co-cultured together both with or without *C. difficile* (always over 70%). Interestingly, platelets were not only attached to macrophages surface but they also localized intracellularly. In contrast to our findings, Trotta and col. observed that platelets surrounded specifically *B. abortus* infected monocytes/macrophages (Trotta et al., 2018). On the other hand and in line with us, Carestia and col. identified platelets/macrophages associations both in LPS presence or absence (Carestia et al., 2019). Altogether, the previous reports and our results reveal platelets versatility, acting both as pathogens patrollers and as links to the immune system.

Platelets and platelets-secreted PAF induce the endocytosis of *B. abortus* (Lee et al., 2016; Trotta et al., 2018) and *S. aureus* (Ali et al., 2017) by monocytes and macrophages. In line with this, we demonstrated that human platelets promote *C. difficile* internalization. It has been proposed that platelets act as carriers of *B. abortus*, delivering bacteria to monocytes/macrophages (Trotta et al., 2018). Unlike this report, we found an increment in macrophages uptake capacity when platelets were pre-incubated with *C. difficile* and then added to macrophages culture as well as when platelets were cultured with *C. difficile* and macrophages together. These results suggest that platelets modulate macrophages ability rather than being transporters of *C. difficile.* Our experiments with live NAP1/BI/027, live CD160 or *CD*H plus the NAP1/BI/027 secretome showed no differences when comparing with *CD*H stimulation, suggesting that the bacteria are not releasing factors that modify the interactions between macrophages and platelets. The obtained results might us speculate that platelets-macrophages physical contact is required to enhance *C. difficile* internalization. Nevertheless, we cannot rule out the possibility that platelets are releasing soluble factors that activate macrophages in a very early way. Although the underlying mechanisms may differ, the outcome is the same: the enhancement of the uptake potential.

To date, it seems that platelets participation against bacteria contribute to develop a protective response as well as to spread infection. Platelets were proposed as rapid defenders against infections mediated by blood-borne pathogens such as *B. cereus* and *S. aureus* (Wong et al., 2016; Wuescher et al., 2016) and a beneficial role for the host has also been suggested in Brucellosis (Trotta et al., 2018; Trotta et al., 2020). On the contrary, *S. pyogenes* dissemination has been related to the presence of platelets (Kahn et al., 2013). Our confocal micrographs show what appears to be processed *C. difficile* in the presence of platelets, suggesting that the interaction of platelets with macrophages could lead to bacteria clearance. Nevertheless, whether the bacteria are destroyed and whether platelets could promote *C. difficile* elimination deserve further investigation.

Different mechanisms can be explored to internalize pathogens. Macropinocytosis, a clathrin-independent endocytic process that internalizes extracellular fluid, nutrients and antigens, has been involved in the uptake of several infectious agents by a broad variety of cell types (Young et al., 1989; Watarai et al., 2001; Niebuhr et al., 2002; Watarai et al., 2002; Mercer and Helenius, 2009; García-pérez et al., 2012; de Carvalho et al., 2015; Nam et al., 2017; Chang et al., 2021). *C. difficile* seems not to be an exception since here we showed that only amiloride (an ion exchange inhibitor) reduced the percentage of *C. difficile*-internalizing macrophages. Amiloride is a selective Na+/H+ antiport inhibitor but how it inhibits macropinocytosis is not yet fully understood. Some proteins of Rho family GTPases (e.g. Rac1 and Cdc42) have been implicated during macropinocytosis (Koivusalo et al., 2010). PLC-PKC-Nox2 pathway has also been described to regulate macropinosome formation, membrane ruffling and fluid phase uptake (Espinal et al., 2022) while Glycogen synthase kinase 3 (GSK3) was proposed as negative regulator by altering Wnt-β-catenin signaling (Tejeda-muñoz et al., 2019; Albrecht et al., 2020). When amiloride was added in the presence of platelets, the reduction in the internalization capacity did not reach basal levels, suggesting that other mechanisms different to macropinocytosis could have a role in *C. difficile* uptake. Likewise, this could also indicate that the presence of platelets per se could decrease the effect of amiloride exerted on macrophages. Importantly, macrophages can play constitutive macropinocytosis, which could serve as an ideal means for delivering *C. difficile* antigens to intracellular PRRs. This is especially relevant at sites of continuous contact with non-self material such as the gut (Canton, 2018), highlighting the relevance of macropinocytosis during CDI.

To the best of our knowledge, this is the first study elucidating the role of human macrophages and platelets in the immune response against *C. difficile.* Although our assays were performed *in vitro*, they provide the basis for proposing platelets as sentinels and modulators of the immune response during CDI. Conventionally, endocytic processes are efficient ways to eliminate invading pathogens, but pathogens can also develop strategies to perpetuate the infection. So many questions still required to be answered. Is *C. difficile* destroyed within macrophages? Are platelets promoters of macrophages microbicidal functions? or do they induce a *C. difficile* reservoir? Is *C. difficile* processed and presented to activate T cells? Can platelets directly eliminate *C. difficile*? To unravel these, further studies are required.

In conclusion, we evidenced that vegetative form of *C. difficile* is internalized by human macrophages. Platelets could directly interact with monocytes and macrophages as well as with bacteria. Moreover, *C. difficile* induced the establishment of monocytes-platelets complexes. Platelets also modulated *C. difficile* uptake mediated by macrophages, enhancing the internalization capacity of the phagocytes. We finally determined that the uptake of *C. difficile* was mainly mediated by macropinocytosis.

These results highlight the role of innate immune cells in CDI, describing new cellular mechanisms involved in the internalization of *C. difficile*. Furthermore, we put in discussion the importance of macropinocytosis in pathogens sensing, a role that is ordinarily underappreciated in the surveillance mechanisms of innate immune cells.

## Data availability statement

The original contributions presented in the study are included in the article/**Supplementary Material**. Further inquiries can be directed to the corresponding authors.

## Ethics statement

The studies involving humans were approved by Ethics Committee of UNNOBA (COENOBA). The studies were conducted in accordance with the local legislation and institutional requirements. The participants provided their written informed consent to participate in this study.

## Author contributions

AB and VP conceived and designed the experiments. AB performed the experiments with RHDP contribution. AB, and FF analyzed the data. PB and RHDP helped with the discussion and the experimental design. AB and VP wrote the first draft of the manuscript. All authors contributed to manuscript reading and revising and approved the submitted version.
