# Supplementary figures and images for: Platelets promote human macrophages-mediated macropinocytosis of Clostridioides difficile
Source: Front Cell Infect Microbiol. 2024 Jan 5;13:1252509. doi: 10.3389/fcimb.2023.1252509 (PMC10796631; doi:10.3389/fcimb.2023.1252509)

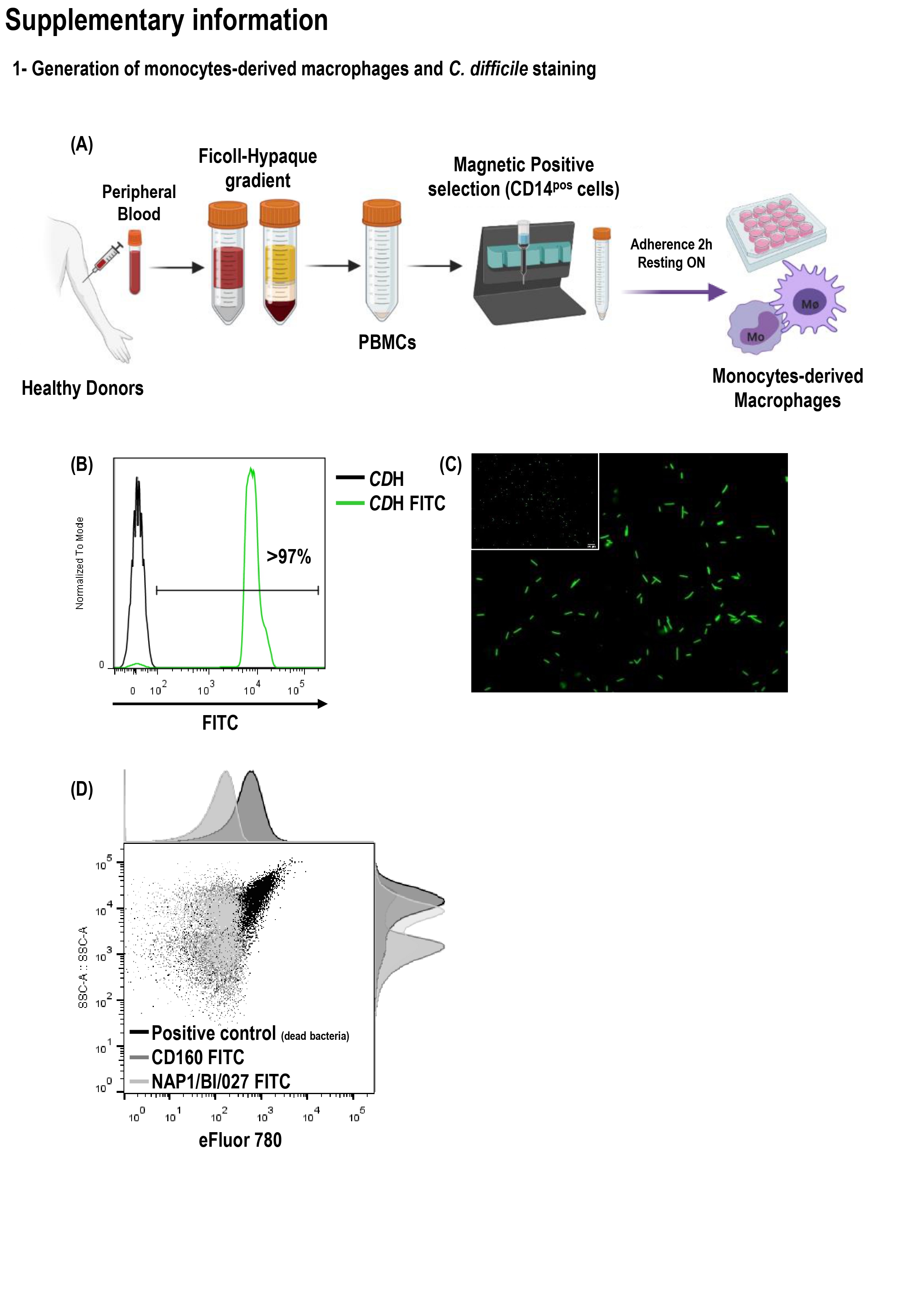

Supplement: Supplementary file 1 [file Image_1.jpeg]

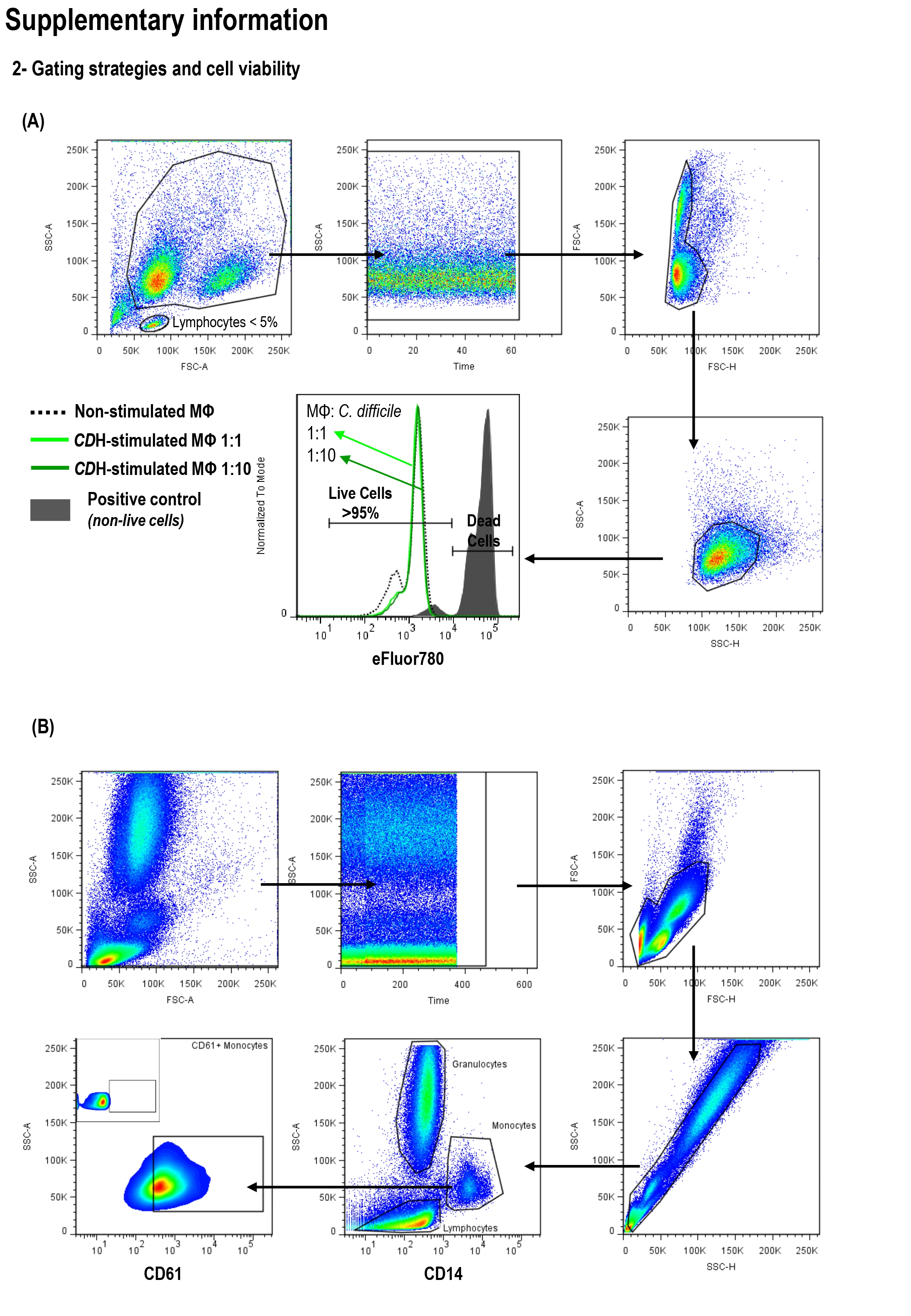

Supplement: Supplementary file 2 [file Image_2.jpeg]

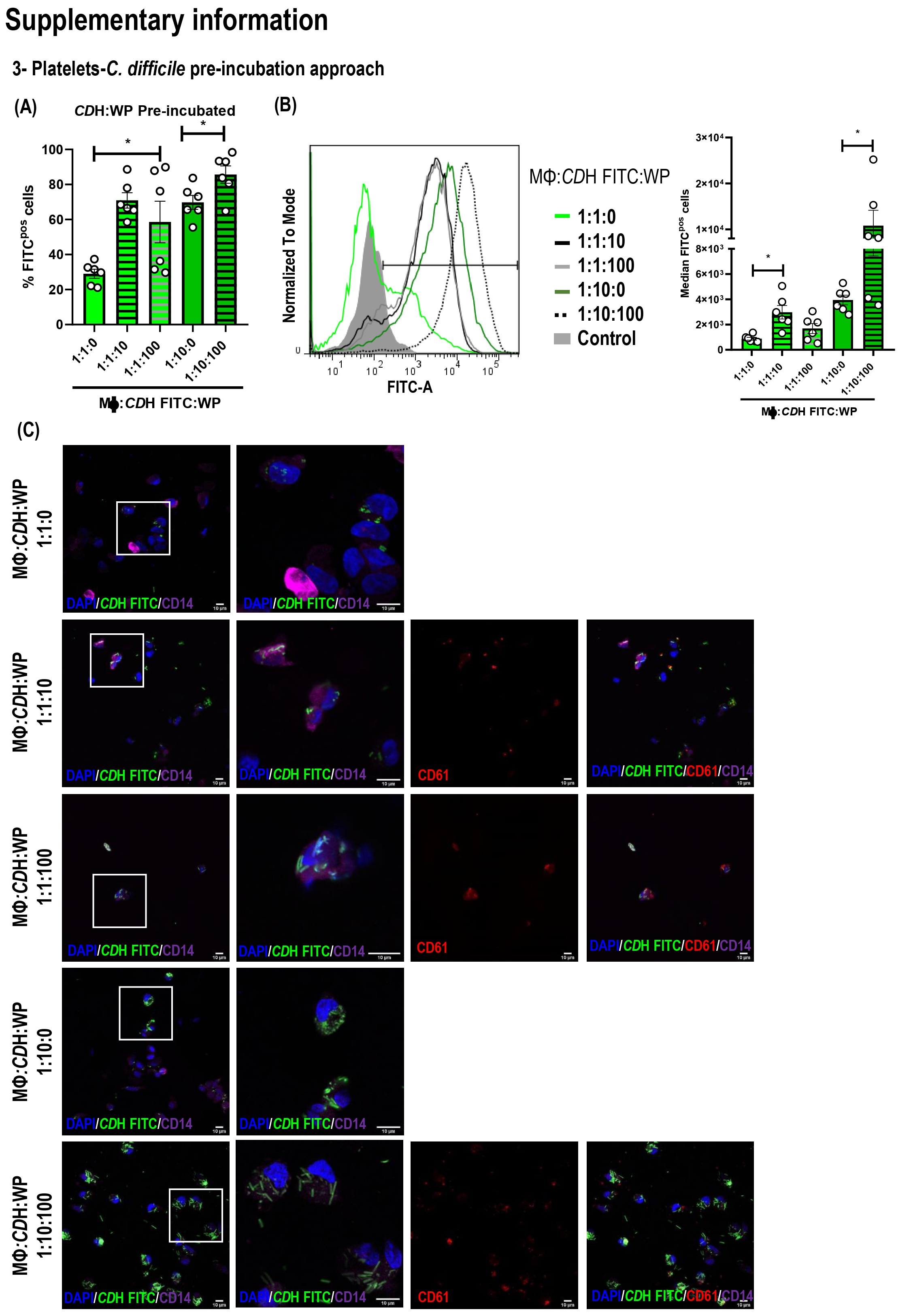

Supplement: Supplementary file 3 [file Image_3.jpeg]

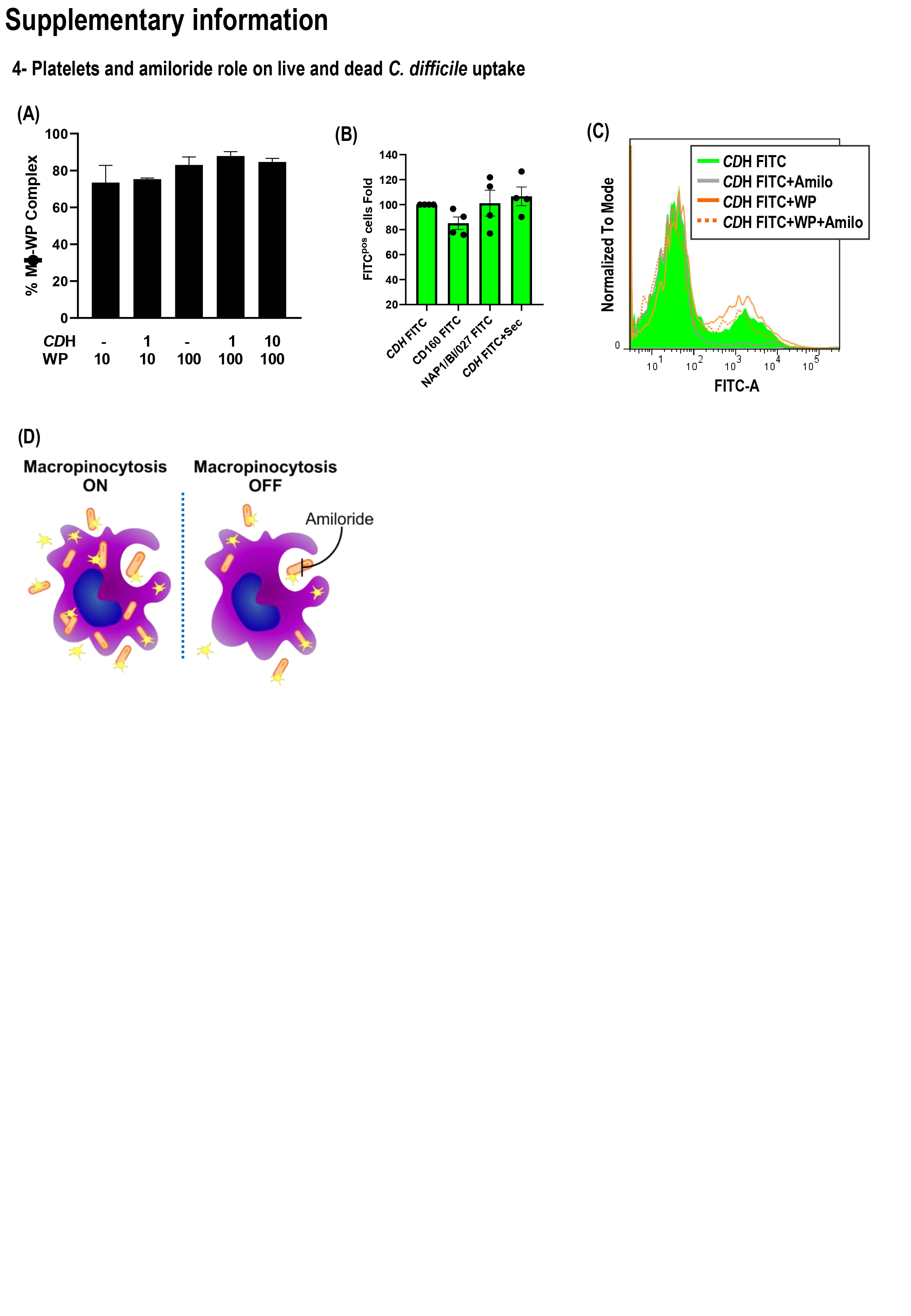

Supplement: Supplementary file 4 [file Image_4.jpg]
