# Supplementary material for: Platelets promote human macrophages-mediated macropinocytosis of Clostridioides difficile
Source: Front Cell Infect Microbiol. 2024 Jan 5;13:1252509. doi: 10.3389/fcimb.2023.1252509 (PMC10796631; doi:10.3389/fcimb.2023.1252509)
Supplement: Supplementary file 5 [file Table_1.pdf]

Supplementary Material

Platelets promote human macrophages-mediated macropinocytosis of *Clostridioides difficile*.

Angela María Barbero[1,2,*], Rodrigo Emanuel Hernández Del Pino[1,2], Federico Fuentes[3], Paula Barrionuevo[3], Virginia Pasquinelli[1,2,*]

*** Correspondence:**

Virginia Pasquinelli virpasquinelli@gmail.com

Angela María Barbero barberoangelamaria@gmail.com

Supplementary figure captions

Supplementary fig. 1 Generation of monocytes-derived macrophages and *C. difficile* staining. A) Peripheral blood was obtained from healthy donors and mononuclear cells (PBMCs) were isolated after Ficoll-Hypaque gradient. Monocytes were sorted from PBMCs by CD14 positive magnetic selection. In all cases, the purity was over 95%. 0.5 x 10^6/ml CD14 positive selected monocytes were cultured for 2h in absence of FBS to promote adherence. Non adherent cells were removed by washing with pre-warmed RPMI and adherent cells were cultured for additional 16-18h (resting ON) in complete media (RPMI 1640 medium supplemented with L-glutamine, 10% FBS, 100 U/mL of Penicillin and 100 µg/mL of Streptomycin). Afterwards, macrophages were stimulated with *C. difficile* and/or co-cultured with platelets. (B-C) Heat-inactivated *C. difficile* (*CD*H) was stained with Fluorescein isothiocyanate (FITC) in carbonate buffer for 2h at 37°C in an orbital shaker. After extensively washing the excess of dye, positive staining was confirmed by flow cytometry (B). Non-stained bacteria were used as control. C) *CD*H FITC was incubated in poly-lysine treated glasses and FITC staining efficiency was evaluated by fluorescence microscopy. D) Live strains of *C. difficile* (non-toxigenic CD160 and hypervirulent NAP1/BI/027) were stained with FITC as stated before. After staining and before using bacteria to infect macrophages, bacterial viability was evaluated by flow cytometry with the FVD eFluor780 dye. A mixture of both heat-killed strains was used as a positive dead control (black).

Supplementary fig. 2 Gating strategies and cell viability. A) Monocyte-derived macrophages population was selected by SSC-A vs. FSC-A strategy. The percentage of residual lymphocytes was evaluated (less than 5% in all experiments). Afterwards, Time parameter was checked to monitor any instrument instability. Cells were then gated to exclude doublets by a double singlets-strategy (FSC-A vs. FSC-H and SSC-A vs. SSC-H). Live versus dead cells gate was determined by employing a viability dye (FVD eFluor780) that irreversibly label dead cells. Heat-killed cells (positive control) were used for compensation controls. Dead cells were excluded from analysis. Representative histograms

showing non-stimulated macrophages and *CD*H-stimulated macrophages (*CD*H= heat-inactivated *C. difficile*) are shown. In all cases, cell viability was over 95%. B) To evaluate monocytes-platelets complexes in fresh blood by flow cytometry, cells were gated as follows: SSC-A vs. FSC-A, SSC-A vs. Time, FSC-A vs. FSC-H and SSC-A vs. SSC-H for doublet exclusion, SSC-A vs. CD14 Alexa Fluor 647 to identify monocytes and, finally, SSC-A vs. CD61 PE to evaluate the percentage of platelets bound to monocytes. A representative density plot with the isotype control at top left is shown.

Supplementary fig. 3 Platelets-*C. difficile* pre-incubation approach. Heat-inactivated *C. difficile* coupled to FITC (*CD*H FITC) was pre-incubated with healthy donors (HD) Washed Platelets (WP) in *CD*H FITC to WP ratios of 1:10, 1:100 and 10:100 for 2h. Then, they were added to HD macrophages (MΦs) culture for 22h at final ratios of 1:1:10, 1:1:100 and 1:10:100 (MΦs: *C. difficile*: WP). The percentage of FITC positive cells (A) and the median intensity of fluorescence (B) were evaluated by flow cytometry. Representative histograms (left) and intensity quantification (right) are shown. In all cases comparisons were done against the experimental conditions without platelets (1:1:0 or 1:10:0). C) MΦs: *CD*H FITC: WP cultures were performed as in A-B and the internalization capacity was evaluated by confocal microscopy. MΦs and WP were detected by direct staining with anti-CD14 (Alexa Fluor 647) and anti-CD61 (PE) antibodies after fixation and permeabilization of the cells. DAPI was used as nuclear counterstaining. The white boxes correspond to the magnified images on the right. Scale bar: 10 μm.

Bars represent the mean ± SEM. A-B, Friedman (1:1:10, 1:1:100 vs 1:1:0) and Wilcoxon (1:10:0 vs 1:10:100) tests. *$p<0.05$, **$p<0.01$

Control= MΦs stimulated with unstained *CD*H.

Supplementary fig. 4 Platelets and amiloride role on live and dead *C. difficile* uptake. A) Macrophages (MΦs) and Washed Platelets (WP) from healthy donors (HD) were co-cultured in the presence or absence of *CD*H for 24h. The formation of MΦs-WP complexes was quantified by flow cytometry. B) MΦs from HD were stimulated with *CD*H FITC or *CD*H FITC plus NAP1/BI/027 secretome (*CD*H FITC+Sec) or infected with CD160 FITC or NAP1/BI/027 FITC for 1h in MΦs:bacteria 1:10 proportion. Percentage of endocytic cells (FITC positive cells) were detected by flow cytometry and relativized to *CD*H FITC-stimulated MΦs condition. Statistical analysis was performed on the raw data. C) MΦs were stimulated with *CD*H FITC in the presence or absence of WP (ratio 1:10:100) for 1h. Amiloride was added 30 minutes before to block macropinocytosis. Median fluorescence intensity of macropinocytic cells was measured by flow cytometry. Representative histograms are shown. D) Summary graphic showing that platelets promote human macrophages-mediated macropinocytosis of *C. difficile.*

(A) Correspond to five individual donors in five independent experiments. (B) Correspond to 4 independent experiments. (A-B) Bars represent the mean ± SEM.

A-B) Friedman test.

Supplementary videos caption

SV1-SV3 correspond to Z-stacks reconstruction from Figure 1F where *C. difficile* internalization by human macrophages is shown. Monocyte-derived macrophages (MΦs) from Healthy Donors were stimulated with heat-inactivated *C. difficile* coupled to FITC (*CD*H FITC). The ratios of MΦs:*CD*H

FITC tested were 1:10 (SV1) for 1h and 1:1 (SV2) and 1:10 (SV3) for 24h assays. Confocal images were taken and analyzed. DAPI is shown in blue, *CD*H FITC in green, F-actin in red, CD14 in purple and DIC (differential interference contrast) in grey. Scale bar: 10 µm

frontiers
